# Effect of High-Intensity Interval Training in Patients With Atrial Fibrillation: A Randomized Clinical Trial

**DOI:** 10.1001/jamanetworkopen.2022.39380

**Published:** 2022-10-31

**Authors:** Jennifer L. Reed, Tasuku Terada, Sol Vidal-Almela, Heather E. Tulloch, Matheus Mistura, David H. Birnie, George A. Wells, Girish M. Nair, Harleen Hans, Kimberley L. Way, Daniele Chirico, Carley D. O’Neill, Andrew L. Pipe

**Affiliations:** 1 University of Ottawa Heart Institute, Ottawa, Ontario, Canada; 2 University of Ottawa, Faculty of Health Sciences, School of Human Kinetics, Ottawa, Ontario, Canada; 3 Deprtment of Medicine, University of Ottawa, Faculty of Medicine, Ottawa, Ontario, Canada; 4 Research Unit on Nutrition and Metabolism, Institut de recherche de l’Hôpital Montfort, Ottawa, Ontario, Canada; 5 Deakin University, School of Exercise and Nutrition Sciences, Institute for Physical Activity and Nutrition, Geelong, Victoria, Australia; 6 Faculty of Kinesiology, University of Calgary, Calgary, Alberta, Canada

## Abstract

**Importance:**

Patients with atrial fibrillation (AF) experience poor functional capacity and quality of life (QOL). High-intensity interval training (HIIT) has been shown to elicit greater improvements in functional capacity and QOL compared with moderate to vigorous intensity continuous training (MICT) in other cardiovascular populations, yet HIIT remains understudied in AF.

**Objective:**

To compare the effects of 12 weeks of HIIT and MICT-based cardiovascular rehabilitation (CR) on functional capacity and general QOL in patients with persistent and permanent AF. Disease-specific QOL, resting heart rate (HR), time in AF, and physical activity (PA) levels were also assessed.

**Design, Setting, and Participants:**

This randomized clinical trial, conducted between November 17, 2015, and February 4, 2020, at a tertiary-care cardiovascular health center in Ottawa, Canada, recruited 94 patients with persistent and permanent AF.

**Interventions:**

High-intensity interval training (23 minutes: two 8-minute interval training blocks of 30-second work periods at 80%-100% of peak power output interspersed with 30-second recovery) or CR (60 minutes: continuous aerobic conditioning within 67%-95% of peak HR and 12-16 of 20 ratings of perceived exertion) twice weekly for 12 weeks.

**Main Outcomes and Measures:**

The primary outcomes were changes in functional capacity (6-minute walk test [6MWT] distance) and general QOL (Short Form 36) from baseline to 12 weeks’ follow-up. Secondary outcomes included changes in disease-specific QOL (Atrial Fibrillation Severity Scale), resting HR, time in AF, and PA levels. An intention-to-treat analysis was used to compare changes between groups.

**Results:**

Of the 94 patients who consented, 86 participated (mean [SD] age, 69 [7] years; 57 [66.3%] men). No significant differences in improvements in 6MWT distance (mean [SD], 21.3 [34.1] vs 13.2 [55.2] m; *P* = .42) and general QOL (Physical Component Summary, 0.5 [6.1] vs 1.1 [4.9] points; *P* = .87) between HIIT and CR were observed. No significant differences in improvements in disease-specific QOL (AF symptoms: −1.7 [4.3] vs −1.5 [4] points, *P* = .59), resting HR (−3.6 [10.6] vs −2.9 [12.4] beats per minute, *P* = .63), and moderate to vigorous PA levels (37.3 [93.4] vs 14.4 [125.7] min/wk; *P* = .35) between HIIT and CR were detected. Participants attended a mean (SD) of 18.3 (6.1) (75.1%) HIIT sessions and 20.0 (4.5) (83.4%) CR sessions (*P* = .36).

**Conclusions and Relevance:**

In this randomized clinical trial, twice-weekly 23-minute HIIT was as efficacious as twice-weekly 60-minute CR in improving functional capacity, general and disease-specific QOL, resting HR, and PA levels in patients with persistent and permanent AF.

**Trial Registration:**

ClinicalTrials.gov Identifier: NCT02602457

## Key Points

**Question:**

In patients with persistent and permanent atrial fibrillation, what is the effect of high-intensity interval training (HIIT) on functional capacity and general quality of life compared with moderate to vigorous intensity continuous training (MICT)?

**Findings:**

In this randomized clinical trial including 86 individuals with atrial fibrillation, HIIT was as efficacious as MICT in improving functional capacity and general quality of life, despite a substantially lower total exercise volume. HIIT was also as efficacious as MICT in improving disease-specific quality of life, resting heart rate, and physical activity levels.

**Meaning:**

In patients with atrial fibrillation, HIIT offers a more time-efficient option to improve physical health and quality of life.

## Teaser

This randomized clinical trial compares the use of high-intensity interval training with moderate to vigorous intensity continuous training to improve functional capacity among individuals with persistent and permanent atrial fibrillation.

## Introduction

Atrial fibrillation (AF), the most common cardiac arrhythmia, is a global epidemic affecting more than 37 million people.^1^ Atrial fibrillation imposes disabling and variable symptoms, a heightened cardiovascular disease risk profile, reduced quality of life (QOL), and increased mortality.^2^ More than 80% of patients report ongoing debilitating AF symptoms^3^ despite pharmacologic and surgical treatments. Persistent and permanent AF are associated with greater patient morbidity and mortality than paroxysmal AF.^4^

Reviews of exercise-based cardiovascular rehabilitation (CR), which primarily includes moderate to vigorous intensity continuous training (MICT), have reported improvements in cardiorespiratory fitness, functional capacity, and QOL, and lower symptom burden, resting and maximal heart rates, and time in AF in patients with persistent and permanent AF.^5,6^ This evidence is, however, limited given the few randomized clinical trials and the heterogeneity of their interventions and outcome measures.

Growing evidence in other cardiovascular disease populations supports the superiority of high-intensity interval training (HIIT) compared with MICT in increasing exercise capacity (including cardiorespiratory fitness^7^ and functional capacity^8^), an independent predictor of subsequent cardiovascular events and mortality.^9,10^ Superior improvements in cardiovascular risk factors following HIIT compared with MICT have also been reported.^11^ In the first trial to examine the impact of HIIT in patients with nonpermanent AF, Malmo and colleagues^12^ reported significant decreases in time in AF, AF symptom frequency, and AF severity, and increases in cardiorespiratory fitness, QOL, and PA levels following HIIT compared with a control group. To our knowledge, no trials have investigated the efficacy of HIIT compared with MICT-based CR in improving the physical health and QOL of patients with persistent and permanent AF. An understanding of the results of different exercise paradigms could enhance exercise prescription among this burgeoning patient population.

The primary purpose of the OPPORTUNITY randomized clinical trial was to compare the effects of a 12-week program of HIIT and MICT-based CR on functional capacity and general QOL in patients with persistent and permanent AF. The secondary purpose was to compare the effects of HIIT and MICT-based CR on AF-specific QOL, resting heart rate (HR), time in AF, and PA levels. It was hypothesized that HIIT would be superior to MICT-based CR in improving functional capacity, general and disease-specific QOL, resting HR, time in AF, and PA levels.

## Methods

### Study Design

This single-center, parallel-group, randomized clinical trial was conducted at the University of Ottawa Heart Institute, Ottawa, Ontario, Canada, a tertiary care cardiovascular center. The study is reported in accordance with the Consolidated Standards of Reporting Trials (CONSORT) reporting guideline and template for intervention description and replication checklist.^13^ The protocol was approved by the Ottawa Health Science Network Research Ethics Board (protocol number: 20150427-01H). All patients provided written informed consent; there was no financial compensation. The trial protocol and statistical analysis plan are provided in Supplement 1.

### Recruitment

Patients were recruited between November 17, 2015, and September 25, 2019; the trial was completed February 4, 2020. Potentially eligible patients were approached by clinicians when they were admitted to the University of Ottawa Heart Institute or seen in outpatient clinics. Recruitment also included advertisement on the University of Ottawa Heart Institute public website and distribution of posters to health practices and community centers within the Champlain Local Health Integration Network of Ontario, Canada.

### Patients

Eligible patients (1) had AF confirmed by an electrophysiologist and categorized as persistent (recurrent AF episodes that last >7 days) or permanent (ongoing AF accepted as a permanent rhythm), (2) had rate-controlled AF with a resting HR less than or equal to 110 beats/min, (3) were able to perform a cardiopulmonary exercise test (CPET) to exhaustion, and (4) were 40 years or older. Patients who (1) were participating in routine exercise training (>2 times per week), (2) had unstable angina or uncontrolled diabetes, or (3) had a diagnosis of severe mitral or aortic stenosis or hypertrophic obstructive cardiomyopathy with obstruction were excluded.

### Screening

Study staff documented patients’ age, sex, and medical information. Race and ethnicity information were not collected from the study outset but added as an amendment after the trial was under way. Because those data were incomplete, they were not included in this analysis. Patients completed a medical assessment with a University of Ottawa Heart Institute physician to confirm the diagnosis of AF and appropriateness for participation in the randomized clinical trial.

### Baseline

Demographic and medical information were retrieved from clinical databases. Participants completed 2 baseline visits. During the first visit, participants underwent a CPET on an electronically braked cycle ergometer (COSMED) to determine the peak aerobic power, HR, and power output. During the second visit, study staff measured patients’ height, body mass, waist circumference, percentage of body fat (using bioelectrical impedance), and resting blood pressure and HR; oversaw the completion of a 6-minute walk test (6MWT); and administered study questionnaires. Each participant was fitted with a 24-hour Holter monitor and 7-day ActiGraph GT3X accelerometer (ActiGraph LLC).

### Randomization

Patients were then randomized in a 1:1 ratio to HIIT or CR using a balanced block, stratified, random sequence computer-generated program by the University of Ottawa Heart Institute Cardiovascular Research Methods Centre. Treatment assignments were placed in sealed, numbered opaque envelopes to ensure concealment. The randomization was stratified by AF classification.

### Intervention

All patients participated in supervised exercise training sessions (ie, HIIT or CR) twice weekly for 12 weeks in our Cardiac Prevention and Rehabilitation Centre. The HIIT and CR sessions were performed at separate times and by different staff to avoid contamination between groups.

### High-Intensity Interval Training

Each HIIT session was 23 minutes in duration and consisted of (1) a 2-minute warm-up at 50% of peak power output; (2) two 8-minute interval training blocks of 30-second work periods at 80% to 100% of peak power output interspersed with 30-second active recovery (16-minute conditioning phase), and 4 minutes of recovery between the blocks; and (3) a 1-minute cooldown at 25% of peak power output on an upright cycle ergometer. Beginning with 50% of the participants’ peak power output, the workload was gradually increased by 10% after every other session until patients were able to exercise at 80% to 100% of their peak power output throughout an exercise training session. To adjust for improvements in exercise tolerance over the 12 weeks, patients were able to increase to 150% of their peak power output. Exercise adherence was assessed by the number of exercise classes participants attended. Exercise compliance was assessed by the number of participants who achieved the prescribed exercise intensity targets (ie, HIIT or MICT).

### Cardiovascular Rehabilitation

Each session was 60 minutes in duration and followed CR guidelines consisting of (1) a 10- to 15-minute warm-up of aerobic exercise; (2) 30 minutes of continuous aerobic conditioning (ie, walking or jogging, cycling, elliptical, rowing) at moderate to vigorous intensity; and (3) a 15-minute cooldown of strengthening and stretching exercises.^14^ Patients were instructed to keep their HRs within 67% to 95% HR peak and encouraged to attain ratings of perceived exertion of 12 to 16 (somewhat hard to hard).

### Follow-up

Within 1 week of completing the 12-week intervention, patients repeated the baseline measures (except for a CPET). Research staff blinded to treatment allocation collected follow-up data.

### Primary Outcomes

#### Change in Functional Capacity

Patients were instructed to walk on an indoor track as far as possible for 6 minutes. Total walking distance was measured in meters. A suggested minimal clinically important difference for the 6MWT is an increase of 54 m.^15^ This valid, practical, and cost-effective tool was selected because almost all CR programs can use it, thus enhancing the generalizability of results.

#### Change in General Quality of Life

General QOL was measured using the Short Form 36, version 1.0, a thoroughly validated survey that has been used in patients with AF.^5,6^ The Short Form 36 yields 8 domains of functional health and well-being scores as well as psychometrically based Physical Component Summary and Mental Component Summary scores. The suggested minimal clinically important difference for the Physical Component Summary and Mental Component Summary is an increase of 5 or more points.^16^

### Secondary Outcomes

#### Change in Disease-Specific QOL

Disease-specific QOL was measured using the Atrial Fibrillation Severity Scale. This scale is a 19-item self-report validated questionnaire that assesses 4 categories: (1) global well-being, (2) AF burden, (3) health care use, and (4) AF symptom score in the previous 4 weeks.^17^

#### Change in HR, Time in AF, and PA Levels

Time in AF was measured over a 24-hour period using a Holter monitor (DR200/HE Holter and Event Recorder, NorthEast Monitoring Inc). Physical activity was measured using an accelerometer (ActiGraph GT3X) at baseline and during the last week of the intervention to capture the PA levels during the intervention. Using previously published protocols,^18^ triaxial cut points were used to define light (150-2689 cpm), moderate (2690-6166 cpm), and vigorous (≥6167 cpm) intensity PA.^19^ Weekly total minutes of PA were used to quantify whether patients were meeting the Canadian Cardiovascular Society exercise targets (≥200 min/wk at moderate intensity) for patients with AF.^2^

### Sample Size Calculation

We used GPower 3.1.9.2 (Universität Kiel) to calculate the sample size to test the 2 primary research hypotheses simultaneously using analysis of variance with repeated measures. A sample size of 74 (37 per group) was needed to detect a conservative effect size (*f*) of 0.32 on the 6MWT and a sample size of 39 (20 per group) was needed to detect an effect size (*f*) of 0.5 on the Physical Component Summary of the Short Form 36 from baseline to 12 weeks’ follow-up between the groups with a 2-sided *P* = .025 significance level and 80% power.^20,21,22^ The larger of these 2 sample size calculations was selected to ensure adequate power. We adjusted our sample size upward to account for an expected 20% dropout rate. We therefore planned to recruit 94 patients in this trial.

### Statistical Analysis

Statistical analyses were conducted with SPSS, version 28 (IBM SPSS Statistics). Baseline characteristics and outcome variables were compared between dropouts and completers. An intention-to-treat analysis was used to compare changes between groups. A 2-step approach for transforming continuous nonnormally distributed variables to normal was applied to applicable baseline and/or follow-up values.^23^ The rank case procedure was applied to variables that remained nonnormally distributed following this approach. We performed a linear mixed-effects model with repeated measures over time (from baseline to week 12) to examine the main effects for time, group, and time × group interaction. The maximum likelihood estimation method to handle missingness and an unstructured covariance matrix were used.

χ^2^ Analyses were used to compare the proportion of patients meeting the minimal clinically important differences, the Canadian Cardiovascular Society exercise targets, and exercise intensity targets between groups. Mann-Whitney tests were used to examine the differences in number and percentage of exercise sessions attended and independent *t* tests were used to analyze ratings of perceived exertion and exercise HRs between groups. Pearson correlation analyses were used to examine the association between changes in HR or time in AF and QOL or symptom-burden values. Nonnormalized values are presented in the Results section for descriptive purposes. Data are reported as mean (SD). Two-sided *P* < .05 was considered significant.

## Results

**Figure 1.**
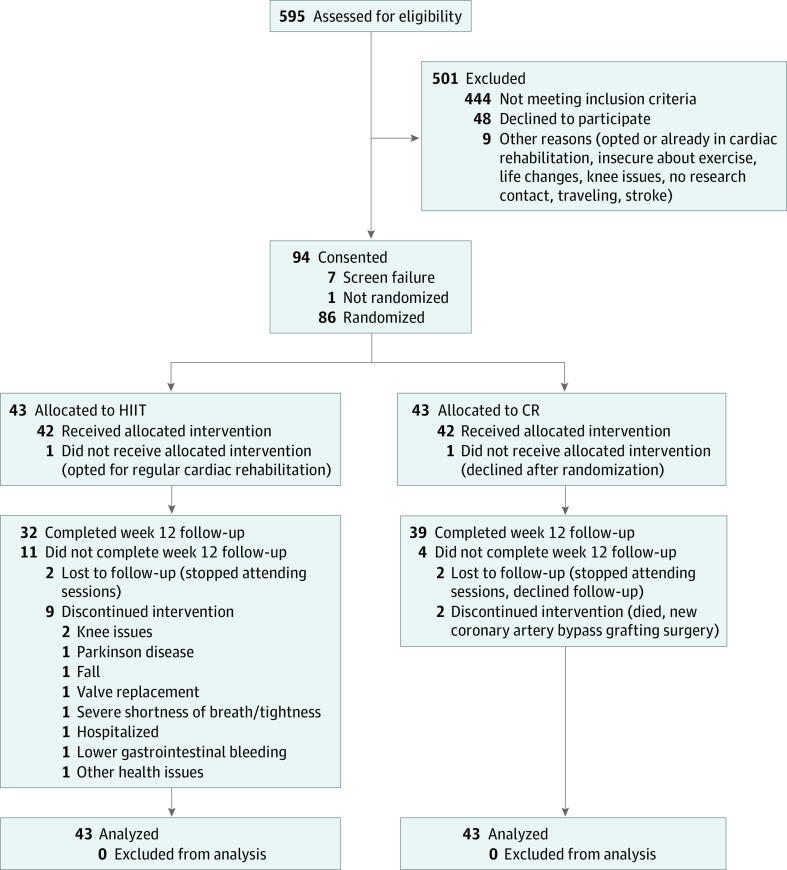
Consolidated Standards of Reporting Trials Flow Diagram of Patients Recruited and Reasons for Withdrawals CR indicates cardiovascular rehabilitation; HIIT, high-intensity interval training.

Of the 595 patients screened, 94 were eligible and consented to participate; 43 patients were randomized to each intervention group (Figure 1). Of the 86 individuals who participated, 57 (66.3%) were men and 29 (33.7%) were women; mean (SD) age was 69 (7) years. Patients’ demographic characteristics, anthropometrics, medical conditions, and medications are presented in Table 1.

**Table 1.** Participant Characteristics at Baseline

Variable	Participants, No. (%)	
	HIIT (n = 43)	CR (n = 43)
Demographic characteristics		
Age, mean (SD), y	68 (8)	71 (7)
Sex		
Female	14 (32.6)	15 (34.9)
Male	29 (67.4)	28 (65.1)
Atrial fibrillation subtype		
Persistent	17 (39.5)	17 (39.5)
Permanent	26 (60.5)	26 (60.5)
Peak aerobic power (V̇o_2_), mean (SD), mL/kg/min	17.9 (5.5)	18.0 (5.6)
Anthropometrics and hemodynamics, mean (SD)		
Height, m	173.5 (8.8)	171.4 (10.1)
Body mass, kg	93.0 (19.5)	88.4 (22.1)
BMI	30.9 (5.7)	29.9 (6.2)
Waist circumference, cm	103.8 (15.6)	100.9 (15.8)
Body fat, %	30.6 (8.3)	29.6 (8.0)
Resting blood pressure, mm Hg		
Systolic	123.8 (18.3)	127.5 (15.6)
Diastolic	77.2 (11.2)	79.6 (9.4)
Resting heart rate, beats/min	73.8 (12.5)	71.5 (14.1)
Medical conditions		
Hypertension	25 (58.1)	23 (53.5)
Dyslipidemia	12 (27.9)	14 (32.6)
Coronary artery disease	7 (16.3)	3 (7.0)
Obstructive sleep apnea	5 (11.6)	4 (9.3)
Diabetes	4 (9.3)	6 (14.0)
Transient ischemic attack	4 (9.3)	0
Stroke	0	4 (9.3)
Congestive heart failure	2 (4.7)	5 (11.6)
Valvular disease	2 (4.7)	1 (2.3)
Valve replacement	2 (4.7)	2 (4.7)
Myocardial infarction	2 (4.7)	2 (4.7)
Angina	1 (2.3)	1 (2.3)
Peripheral vascular disease	1 (2.3)	0
Ischemic cardiomyopathy	0	3 (7.0)
Medications		
Anticoagulant	38 (88.4)	38 (88.4)
β-Blocker	29 (67.4)	26 (60.5)
Statin	17 (39.5)	20 (46.5)
Calcium antagonist	15 (34.9)	9 (20.9)
ACE inhibitor	13 (30.2)	8 (18.6)
Diuretic	11 (25.6)	10 (23.3)
Digoxin	7 (16.3)	5 (11.6)
Aspirin	6 (14.0)	3 (7.0)
ARB	6 (14.0)	11 (25.6)
Nitrate	5 (11.6)	4 (9.3)
Thyroid hormone	4 (9.3)	8 (18.6)
Clopidogrel	3 (7.0)	0
Hypoglycemic	2 (4.7)	3 (7.0)
Antidepressant	2 (4.7)	3 (7.0)
Antiplatelet	1 (2.3)	0
Antiarrhythmic	1 (2.3)	4 (9.3)

Abbreviations: ACE, angiotensin-converting enzyme; ARB, angiotensin receptor blocker; BMI, body mass index (calculated as weight in kilograms divided by height in meters squared); CR, cardiovascular rehabilitation; HIIT, high-intensity interval training; V̇o_2_, aerobic power.

A total of 15 patients (16%) were lost to follow-up or dropped out of the trial (Figure 1); a greater number of dropouts were observed in the HIIT (n = 11) than CR (n = 4) group (*P* = .047). There were no significant differences in baseline characteristics between noncompleters in the HIIT and CR groups, or between all noncompleters and completers.

### Six-Minute Walk Test

The impact of the exercise interventions on patients’ functional capacity is presented in Table 2. A significant main effect of time showed an increase in 6MWT distance (21.3 [34.1] vs 13.2 [55.2] m; *F* = 10.494; *P* = .002). No significant main effect of group (*F* = 0.066; *P* = .80) or time × group interaction (*F* = 0.652; *P* = .42) was observed. Six patients (19%) in the HIIT group and 8 (21%) in the CR group achieved the suggested minimal clinically important difference (ie, 54 m); no significant differences in these proportions were observed (χ^2^ = 0.035; *P* = .85).

**Table 2.** Participants’ Physical Health and Quality of Life at Baseline and Change Scores

Variable	Mean (SD)				*P* value		
	HIIT (n = 43)		CR (n = 43)		Time effect	Group effect	Time × group effect
	Baseline	Change	Baseline	Change			
Functional capacity							
6MWT, m	523.6 (88.5)	21.3 (34.1)	522.3 (121.0)	13.2 (55.2)	.002	.80	.42
General quality of life: SF-36^a^							
Physical functioning	41.8 (9.2)	1.9 (5.8)	43.8 (8.8)	2.7 (6.2)	.003	.56	.75
RL–physical health	41.7 (11.3)	2.9 (9.3)	42.2 (12.2)	0.6 (11.1)	.69	.52	.20
Bodily pain	38.5 (7.0)	−1.5 (9.0)	37.3 (7.3)	−1.5 (8.5)	>.99	.37	.89
General health	44.5 (9.2)	0.7 (6.1)	42.5 (10.0)	1.4 (7.2)	.11	.40	.42
Vitality	45.6 (10.5)	4.4 (10.2)	45.6 (9.3)	3.1 (8.7)	.05	.59	.22
Social functioning	49.2 (10.4)	2.2 (6.1)	49.4 (9.6)	0.9 (6.7)	.90	.46	.27
RL–emotional problems	48.2 (11.2)	2.6 (10.1)	51.4 (9.2)	0.8 (11.3)	>.99	.26	.29
Mental health	51.8 (8.7)	2.4 (8.1)	52.4 (7.6)	−0.4 (6.1)	.99	.49	.19
Physical Component Summary	38.4 (7.1)	0.5 (6.1)	37.9 (8.8)	1.1 (4.9)	.14	.96	.87
Mental Component Summary	53.0 (10.3)	2.8 (8.4)	53.4 (10.7)	−0.2 (7.6)	.04	.57	.08
AF-specific quality of life: AFSS^b^							
Global well-being (range, 1-10)	7.6 (1.7)	0.1 (1.0)	6.8 (2.4)	0.4 (1.3)	.59	.16	.33
Frequency of AF (range, 0-10)	1.4 (0.8)	0.3 (0.7)	1.8 (1.6)	0.0 (1.6)	.55	.78	.17
Duration of AF (range, 1-10)	1.7 (1.8)	1.0 (2.2)	2.0 (2.1)	0.1 (2.2)	.82	.87	.18
Severity of AF (range, 1-10)	3.8 (2.2)	−0.3 (1.7)	3.9 (2.5)	−0.4 (1.9)	.76	.91	.79
Total burden (range, 0-30)	22.6 (3.3)	−1.8 (3.6)	21.4 (4.4)	−0.4 (3.4)	.83	.53	.10
AF symptoms (range, 0-35)	8.1 (5.5)	−1.7 (4.3)	8.5 (5.9)	−1.5 (4.0)	.004	.86	.59
Visits to the ED	0.2 (0.6)	0.0 (0.2)	0.5 (0.9)	0.0 (0.8)	.70	.14	.27
Hospitalizations	0.1 (0.3)	0.0 (0.2)	0.3 (0.7)	−0.2 (0.5)	.50	.14	.21
Time in AF, %^c^	93.8 (15.7)	0.1 (0.5)	98.1 (4.8)	−6.2 (23.2)	.70	.67	.07
Physical activity levels							
Light							
Min/wk	604.8 (434.3)	218.3 (407.3)	627.9 (488.4)	50.2 (528.4)	.02	.63	.44
% Per wk^d^	6.0 (4.3)	0.9 (5.5)	6.2 (4.8)	0.5 (5.6)	.02	.63	.44
Moderate							
Min/wk	109.0 (86.9)	30.3 (91.6)	123.7 (108.7)	9.0 (122.0)	.04	.93	.82
% Per wk	1.1 (0.9)	0.2 (1.0)	1.2 (1.1)	0.1 (1.3)	.03	.68	.37
Vigorous							
Min/wk	5.7 (7.4)	1.2 (9.6)	5.7 (7.0)	0.0 (6.1)	.49	.75	.48
% Per wk	0.1 (0.1)	0.0 (0.1)	0.1 (0.1)	0.0 (0.1)	.49	.75	.48
MVPA							
Min/wk	114.7 (87.6)	37.3 (93.4)	129.4 (109.3)	14.4 (125.7)	.01	.62	.35
% Per wk	1.1 (0.9)	0.2 (1.0)	1.2 (1.1)	0.2 (1.3)	.01	.62	.35
Anthropometrics and hemodynamics							
Body mass index^e^	30.9 (5.7)	−0.1 (1.0)	29.9 (6.2)	−0.3 (0.9)	.44	.35	.71
Waist circumference, cm	103.8 (15.6)	−0.4 (4.0)	100.9 (15.8)	−0.9 (4.2)	.17	.24	.59
Fat mass, %	30.6 (8.3)	−0.4 (4.1)	29.6 (8.0)	−0.7 (3.6)	.27	.50	.74
Resting blood pressure, mm Hg							
Systolic	123.8 (18.3)	1.1 (14.9)	127.5 (15.6)	−1.8 (11.8)	.78	.46	.51
Diastolic	77.2 (11.2)	−0.3 (11.2)	79.6 (9.4)	−2.7 (7.0)	.17	.48	.37
Resting heart rate, beats per min	73.8 (12.5)	−3.6 (10.6)	71.5 (14.1)	−2.9 (12.4)	.01	.53	.63

Abbreviations: AF, atrial fibrillation; AFSS, Atrial Fibrillation Severity Scale; CR, cardiovascular rehabilitation; ED, emergency department; HIIT, high-intensity interval training; 6MWT, 6-minute walk test; MVPA, moderate to vigorous physical activity; RL, role limitations; SF-36, Short Form 36.

^a^ Higher scores denote better perceived health, except for bodily pain.

^b^ Higher well-being scores; lower AF burden; fewer visits to the emergency department and hospitalizations, and lower AF symptom scores denote better perceived health.

^c^ Measured by 24-hour Holter monitor.

^d^ The percentage of time spent in light, moderate, vigorous, and MVPA was calculated to account for differential wear time across patients.

^e^ Calculated as weight in kilograms divided by height in meters squared.

### Quality of Life

The impact of the exercise interventions on patients’ general and AF-specific QOL scores are reported in Table 2. Significant main effects of time showed improvements in physical functioning (*F* = 9.476; *P* = .003) and Mental Component Summary (*F* = 4.413; *P* = .04) scores. Nine patients (29%) in the HIIT group and 9 (24%) in the CR group achieved the minimal clinically important difference (ie, ≥5 points) for the Physical Component Summary score; no significant differences in these proportions were found (χ^2^ = 0.192; *P* = .66). Nine patients (29%) in the HIIT group and 6 (16%) in the CR group achieved the minimal clinically important difference (ie, ≥5 points) for the Mental Component Summary score; no significant differences in these proportions were observed (χ^2^ = 1.611; *P* = .20).

### Change in HR and Time in AF

The impact of the exercise interventions on patients’ HR and time in AF are presented in Table 2. No significant differences in the proportion of patients receiving vs not receiving medications between the HIIT and CR groups were observed at follow-up.

### PA Levels

The impact of the exercise interventions on patients’ PA levels are reported in Table 2. Four patients (11%) in the HIIT group and 9 (24%) in the CR group met the Canadian Cardiovascular Society exercise targets (≥200 min/wk) at baseline; no significant differences between groups were observed (χ^2^ = 2.168; *P* = .14). Ten patients (32%) in the HIIT group and 10 (28%) in the CR group met the Canadian Cardiovascular Society exercise targets at follow-up; no significant differences between groups were observed (χ^2^ = 0.160; *P* = .69).

### Exercise Adherence, Compliance, and Safety

**Figure 2.**
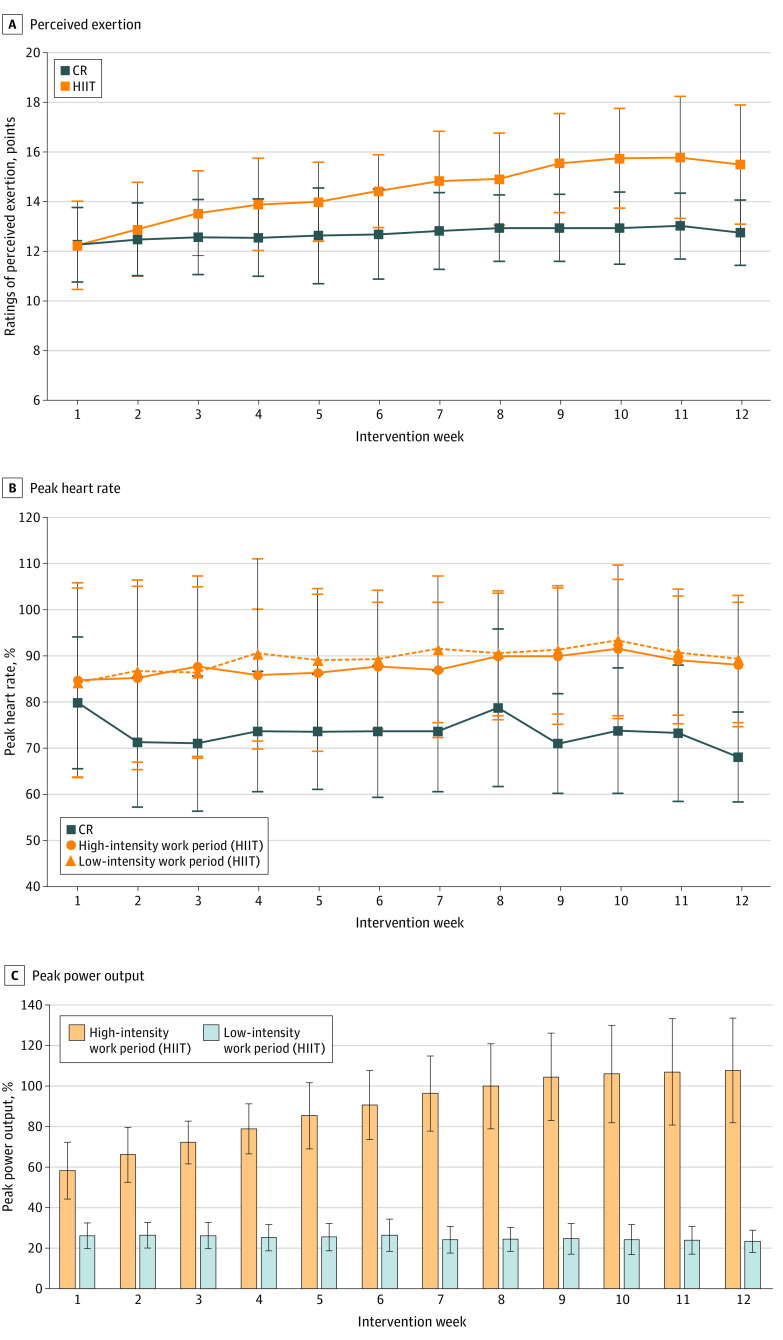
Exercise Training Responses The exercise training responses including (A) ratings of perceived exertion, (B) percentage of peak heart rate, and (C) percentage of peak power output throughout the high-intensity interval training (HIIT) and cardiovascular rehabilitation (CR) groups from intervention week 1 to 12. Values are plotted as means (SDs).

Of the average 24 exercise sessions offered, the HIIT participants attended a mean (SD) of 18.3 (6.1) (75.1%) and the CR participants attended 20.0 (4.5) (83.4%) sessions (*P* = .36). The exercise HRs, power output, and ratings of perceived exertion at which the HIIT and CR groups exercised throughout the program are presented in Figure 2. No significant differences in the proportion of patients who complied with their exercise intensity targets (ie, comparison of actual vs prescribed percentage of peak HR or percentage power output targets) were observed across HIIT (26 [63%]) and CR (29 [73%]) (χ^2^ = 0.767; *P* = .38). Adverse events are reported in Table 3. A total of 2 (0.27%) exercise-related adverse events (ie, nausea/vomiting, knee swelling/medial collateral ligament tear, uncontrolled HR) occurred during the 749 HIIT sessions vs 1 (0.13%) in the 800 CR sessions.

**Table 3.** Participants’ Adverse and Unexpected Events

Events	No.					
	Baseline exercise testing		Follow-up exercise testing		Intervention phase^a^	
	HIIT	CR	HIIT	CR	HIIT	CR
Severe						
Syncope	0	0	0	0	1	0
Fall	0	0	0	0	1	0
Shortness of breath	0	0	0	0	1^b^	1
Angina	0	0	0	0	0	1
Incarcerated left inguinal hernia	0	0	0	0	1	0
Lower gastrointestinal bleeding	0	0	0	0	0	1^b^
Moderate						
Back pain	1 (6MWT)	0	0	0	0	0
Gout	0	0	0	0	1	0
Nausea/vomiting	0	0	0	0	1 (ES)	0
Uncontrolled HR	1 (CPET)	0	0	0	0	1 (ES)
Knee swelling/MCL tear	0	0	0	0	1 (ES)^b^	0
Mild						
Muscular pain (ankle, back, knee)	2 (6MWT)	1 (6MWT); 1 (CPET)		3 (6MWT)	0	0
Dizziness	1 (6MWT)	1 (CPET)	0	0	0	0
Chest pressure	1 (6MWT)	2 (6MWT)	0	0	0	0
Angina	0	1 (CPET)	0	0	0	0
Total	6	6	0	3	7	4

Abbreviations: 6MWT, 6-minute walk test; CPET, cardiopulmonary exercise test; CR, cardiovascular rehabilitation; ES, exercise session; HIIT, high-intensity interval training; HR, heart rate; MCL, medial collateral ligament.

^a^ Adverse events during the intervention phase that were not accompanied by ES represent events outside the ESs.

^b^ Adverse event that led to participant dropout. The severe shortness of breath led to a follow-up examination by a physician who then scheduled the patient for a valve replacement; this procedure led to the participant discontinuing the intervention.

## Discussion

The evidence regarding the functional capacity and QOL benefits of MICT-based CR in patients with persistent and permanent AF is limited, and the efficacy of HIIT in improving such outcomes is understudied. Contrary to our primary hypothesis, superior improvements in functional capacity and general QOL were not observed following twice-weekly 23-minute HIIT compared with 60-minute MICT-based CR sessions for 12 weeks. Superior improvements in disease-specific (ie, AF symptoms), resting HR and PA levels were also not observed.

### Functional Capacity

The patients, regardless of group assignment, achieved improvements (HIIT: 21.3 m vs CR: 13.2 m) in functional capacity following twice-weekly 23-minute HIIT and 60-minute CR sessions. Both groups fared similarly despite the 336-minute difference in total exercise conditioning volume (CR: 720 minutes vs HIIT: 384 minutes). A recent meta-regression analysis in patients with AF reported that exercise sessions of at least 60 minutes could significantly increase 6MWT distance,^24^ yet the patients in the HIIT group of our study demonstrated significant increases after a program duration of only 23 minutes. A meta-analysis showed, however, superior increases of 47 m in 6MWT distance following MICT-based CR compared with control (ie, usual care) in patients with paroxysmal, persistent, and permanent AF.^5^ The less pronounced improvements observed in our study (HIIT: 21 m vs CR: 13 m) may reflect the patients’ higher-than-expected functional capacities; this also was surprising, especially given our exercise training (>2 times per week) exclusion criterion. It is also possible that a longer intervention duration may have produced larger treatment effects. Our findings are nonetheless important because greater distances have been associated with fewer limitations in activities of daily living,^25^ which are important patient-reported outcomes.^26^

### Quality of Life

Quality of life following HIIT is infrequently reported,^27^ leaving uncertainty regarding the effects of HIIT on this patient-reported outcome. Martland et al,^27^ in their meta-review of HIIT outcomes, noted that approximately 25% of patients with cardiometabolic disorders experienced improvements in general and disease-specific QOL following HIIT compared with controls. We observed significant improvements in overall Mental Component Summary scores (mean [SD], 1.2 [8.1] points) from baseline to following 12 weeks of HIIT and CR. These increases appear to be smaller than those reported by others,^5^ which may reflect our higher baseline values and shorter intervention duration (ie, 12 weeks vs 6 months^28^). Consequently, less than 30% of patients achieved the suggested minimal clinically important difference for the Physical Component Summary and Mental Component Summary. A decrease in the Atrial Fibrillation Severity Scale symptom scores was observed over time following HIIT and CR. Systematic reviews have noted that few studies have examined changes in Atrial Fibrillation Severity Scale scores following exercise training in patients with AF, permitting only limited comparisons.^5,6^

### HR and Time in AF

We observed reductions in resting HR (mean change: −3 beats/min) from baseline to following 12 weeks of HIIT and CR. Pooled data from 3 trials in patients with nonpermanent and permanent AF have similarly noted decreases in resting HR (mean difference: −4.61 beats/min; *P* = .001) in exercising vs control participants.^5^ Greater HR control in AF has been shown to improve QOL and symptom burden.^29^ We did not observe any significant correlations between changes in resting HR or time in AF (percent) and QOL or symptom-burden values (*P* > .05). This nonsignificance may be a result of most patients having permanent AF and being in AF 97% [11%] of the time.

### PA Levels

Few patients randomized to HIIT or CR met the Canadian Cardiovascular Society exercise target at baseline (<25%) or 12 weeks’ follow-up (<33%),^2^ yet we observed significant increases in light, moderate, and moderate to vigorous intensity PA levels (minutes per week) at follow-up with no between-group differences. Two studies in patients with AF have reported device-measured PA levels.^12,30^ Malmo et al^12^ reported a mean (SD) increase in moderate intensity (30 [80] min/d) and vigorous intensity (7 [17] min/d) PA following 12 weeks of HIIT; Borland et al^30^ did not observe increases in moderate to vigorous PA following 12 weeks of MICT-based CR (mean [SD], 164 [133] to 134 [103] min/wk; *P* > .05). The benefits of regular PA for patients with AF are innumerable^6^ and our findings provide insight into the potential interchangeability of our HIIT and MICT-based CR protocols as a mode of exercise for increasing daily moderate to vigorous PA.

### Exercise Adherence, Compliance, and Safety

Although the programs differed in duration, modalities, and intensities, the exercise offerings were, overall, highly attended (79% [>70% is considered high adherence]^31^) and safe with no significant differences in compliance between the groups. Lack of time remains a commonly cited barrier to regular exercise participation even among patients with cardiovascular disease,^32^ in whom insufficient PA predicts premature cardiovascular disease burden and mortality.^33^ Our more intense and time-efficient HIIT sessions may be advantageous because they produced similar improvements in functional capacity, general (ie, Mental Component Summary) and disease-specific (ie, AF symptoms) QOL, resting HR, and PA levels (ie, light and moderate to vigorous intensity) compared with CR.

### Limitations

This study has limitations; it was a single-center randomized clinical trial. Replication of this study at other sites is needed to confirm our findings. Although cardiopulmonary exercise testing is the standard for assessing exercise capacity, we used the 6MWT. This test is a valid, practical, and cost-effective tool of exercise capacity that almost all CR programs can use. Our sample involved predominately men (66.3%), which may further limit the generalizability of our findings. Previous studies exploring exercise training in adults with AF also have mainly involved men (<25% women).^34^ Although we were successful in recruiting 33.7% (n = 29) women, we did not purposedly recruit by sex. Future studies should aim to recruit an equal number of men and women and perform appropriately powered sex-based analyses. Because some CR participants exercised at the higher end of their 67% to 95% HR peak exercise prescription range, this may have affected the ability to detect differences in physical and mental health outcomes between the HIIT and MICT groups. In addition, we performed several statistical tests across our outcomes, which may have increased the chance of detecting statistical differences.

## Conclusions

The findings from this randomized clinical trial reveal similar improvements in functional capacity, general and disease-specific QOL, resting HR, and PA levels in patients with persistent and permanent AF following twice-weekly 23-minute HIIT when compared with 60-minute CR sessions for 12 weeks despite a substantial difference in total exercise conditioning volume of 336 minutes. Both offerings were well attended and safe. Cardiovascular rehabilitation and center-based exercise programs may confidently apply MICT or HIIT to improve the physical health and QOL in patients with persistent and permanent AF who are searching for additional treatment options; however, patients’ susceptibility to health complications (eg, joint injuries) should be considered given the higher dropouts in the HIIT group.

## Notes

Supplement 1.
Trial Protocol

Supplement 2.
Data Sharing Statement
