# Supplementary material for: Effect of High-Intensity Interval Training in Patients With Atrial Fibrillation: A Randomized Clinical Trial
Source: JAMA Netw Open. 2022 Oct 31;5(10):e2239380. doi: 10.1001/jamanetworkopen.2022.39380 (PMC9623436; doi:10.1001/jamanetworkopen.2022.39380)
Supplement: Supplement 1. — Trial Protocol [file jamanetwopen-e2239380-s001.pdf]

Research Protocol

Study title: Exercise Training in Patients with Atrial Fibrillation - Addressing Clinical Needs (OPPORTUNITY Study)

Principal Investigator:
Dr. Jennifer Reed, PhD, MEd CS
Associate Scientist
Division of Prevention and Rehabilitation
University of Ottawa Heart Institute
40 Ruskin Street
Ottawa, ON, K1Y 4W7
Canada

Co-Investigator:
Dr. Girish Nair, MD
Cardiologist
University of Ottawa Heart Institute
40 Ruskin Street
Ottawa, ON, K1Y 4W7
Canada

Collaborators:
Dr. Andrew Pipe, CM, MD
Chief
Division of Prevention and Rehabilitation
University of Ottawa Heart Institute
40 Ruskin Street
Ottawa, ON, K1Y 4W7
Canada

Dr. David Birnie, MD
Cardiologist
University of Ottawa Heart Institute
40 Ruskin Street
Ottawa, ON, K1Y 4W7
Canada

Dr. Heather Tulloch, PhD
Psychologist
Division of Prevention and Rehabilitation
University of Ottawa Heart Institute
40 Ruskin Street
Ottawa, ON, K1Y 4W7
Canada

Research Coordinators:
Matheus Mistura, MSc
Division of Prevention and Rehabilitation
University of Ottawa Heart Institute
40 Ruskin Street
Ottawa, ON, K1Y 4W7
Canada

Abstract
Recommended standards of care do not, ironically, include the prescription of exercise to target and ameliorate the progressive health decline and overall feeling of well-being in patients with atrial fibrillation. Recent Standards for the Provision of Cardiac Rehabilitation of Ontario are calling for the consideration for referral and enrollment of patients with atrial fibrillation in cardiovascular rehabilitation – evidence is needed to support and confirm these efforts. The main purpose of this project is to evaluate the effects of high-intensity interval training (HIIT) compared to moderate-intensity continuous exercise training (MICE) on exercise capacity and quality of life in patients with persistent or permanent atrial fibrillation. Positive results from this study will identify a new intervention that: produces important improvements in patient-rated clinical, behavioural and exercise outcomes; provides mechanistic insight into the role of exercise training in the management of atrial fibrillation; requires minimal equipment; and is appealing to patients with atrial fibrillation.

Background
Atrial fibrillation is the most common sustained cardiac arrhythmia, with a lifetime risk of 26% in persons aged 40 years and older. Given an aging population, burgeoning rates of obesity and diabetes, and the growing number of patients who have experienced various forms of heart disease and their complications, the prevalence of atrial fibrillation is expected to increase. The presence of atrial fibrillation substantially increases the risk of stroke, and significantly reduces exercise tolerance (-20%). In fact, declining exercise capacity is often the presenting symptom leading to the diagnosis of this arrhythmia. Mechanisms of exercise intolerance in persons with atrial fibrillation are not well defined but presumed to be due to poor heart rate control, loss of atrioventricular synchrony and declining cardiac output. Excessive heart rate responses to exercise may have deleterious effects on ventricular function further limiting exercise capacity. Persons with atrial fibrillation, because of disordered cardiac function and diminished exercise tolerance, typically report poor quality of life. The restoration of sinus rhythm or the slowing of the rate of atrial fibrillation has been shown to improve symptoms and quality of life. However, adequate heart rate control at rest does not imply adequate control during exercise. Despite the presence of exercise intolerance, poor quality of life, weight gain and an associated decline in overall health among patients with atrial fibrillation, recommended standards of care do not, ironically, include the prescription of exercise to target and ameliorate these significant health issues.

Exercise training is a recognized form of treatment of persons with heart disease. A few studies have demonstrated the beneficial effects of moderate-intensity continuous exercise training in patients with atrial fibrillation. Although demonstrating positive results, these studies have significant limitations including small sample size, lack of follow-up measures, poor study design and high withdrawal rates. A novel exercise paradigm such as high-intensity interval training, a form of exercise in which

participants alternate periods of short, intense, non-oxidative exercise with less intense recovery periods, may be more efficient and motivating, and may help to improve adherence to exercise training in atrial fibrillation patients. A training program that offers a smaller time commitment is advantageous as “lack of time” remains a commonly cited barrier to routine exercise participation even among cardiovascular disease patients. Given the growing number of trials in cardiovascular disease patients demonstrating the safety and superiority of high-intensity interval training compared to moderate-intensity continuous exercise training, investigations determining the type of exercise training intervention most effective in improving clinical, exercise and behavioural outcomes in those with atrial fibrillation are needed. It is likely that high-intensity interval training (compared to moderate-intensity continuous exercise training) will attenuate the rate of heart rate acceleration to a greater degree enabling persons with atrial fibrillation to be more active. Such outcomes would lead to significant improvements in exercise capacity and quality of life. To date, no studies have evaluated the potential benefits of high-intensity interval training in those with atrial fibrillation. The Ontario Standards for the Provision of Cardiac Rehabilitation are calling for such research.

Purpose/Objective and Rationale

The primary objective of this study is to evaluate, in adults with persistent or permanent atrial fibrillation from baseline to 12 weeks follow-up, the effects of moderate-intensity continuous exercise training and high-intensity interval training on exercise capacity and quality of life. Secondary objectives include evaluating the effects of moderate-intensity continuous exercise training and high-intensity interval training on: exercise adherence; activity status; atrial fibrillation-specific quality of life; symptom burden; self-reported symptom frequency and severity; anxiety and depressive symptoms; insomnia severity; sleep apnea risk; and, self reported sleep patterns; heart rate (HR) control; lower body muscular fitness; and exercise capacity measured by cardiopulmonary exercise test (CPET) [in a subset of willing participants].

Hypotheses

In adults with persistent or permanent atrial fibrillation, a 12-week program of high-intensity interval training will be superior to moderate-intensity continuous exercise training in improving: 1) exercise capacity measured by 6-minute walk test distance; and, 2) quality of life measured by the physical component summary (PCS) measure of the Short Form 36 Health Survey Questionnaire (SF-36). Further, a 12 week program of high-intensity interval training will be superior to moderate-intensity continuous exercise training in improving: 1) exercise adherence measured by accelerometer (and verified by the 7-day FITT log; 2) activity status measured by the Duke Activity Status Index (DASI); 3) atrial fibrillation specific quality of life measured by the University of Toronto Atrial Fibrillation Severity Scale (AFSS); 4) symptom burden measured by the Canadian Cardiovascular Society Severity of Atrial Fibrillation (CCS-SAF) scale; 5) self-reported symptom frequency and severity measured by the 7-day symptom diary; 6) anxiety and depressive symptoms measured by the Hospital Anxiety and Depression Scale (HADS); 7) insomnia severity measured by the Insomnia Severity Index; 8) sleep apnea risk measured by the STOP-BANG Sleep Apnea Questionnaire; 9) self reported sleep patterns measured by the 7-day sleep diary; and, 10) HR control measured by 24-hour Holter ECG recordings and ECG recordings at each CPET; 11) lower body muscular fitness measured by standard load tests; and 12) exercise capacity measured by cardiopulmonary exercise test (CPET) [in a subset of willing participants].

Clinical Relevance
Positive results from this study will identify a new intervention that: produces important improvements in patient-related clinical, behavioural and exercise outcomes; provides mechanistic insight into the role of exercise training in the management of atrial fibrillation; requires minimal equipment; and is appealing to patients with atrial fibrillation.

Design
This is a single-centre, randomized controlled trial to evaluate the effects of high-intensity interval training when compared to moderate-intensity continuous exercise training on exercise capacity and quality of life in patients with persistent or permanent atrial fibrillation.

Setting
This trial will be conducted at the University of Ottawa Heart Institute (UOHI). Specifically, all testing and exercise training will be performed in the Cardiac Diagnostic Centre, on Track in the Division of Prevention and Rehabilitation, or the *Goodlife* exercise facilities at The Ottawa Hospital – Civic Campus.

Inclusion and Exclusion Criteria:
Inclusion criteria:
1. persistent or permanent atrial fibrillation;
2. rate controlled with a resting ventricular rate of equal to or less than 110 bpm;
3. able to perform a symptom-limited exercise test;
4. at least 40 years of age;
5. patient agrees to sign informed consent.

Exclusion criteria
1. currently participating in routine exercise training (more than two times per week);
2. unstable angina;
3. uncontrolled diabetes mellitus;
4. diagnosed severe mitral or aortic stenosis;
5. diagnosed hypertrophic obstructive cardiomyopathy with significant obstruction;
6. unable to provide written, informed consent.

Sample Size
The sample is sufficient to test the two primary research hypotheses simultaneously using repeated measures mixed models analyses (group: (1) moderate-intensity continuous exercise training, (2) high-intensity interval training; time: (1) baseline, (2) 12 weeks follow-up). A sample size 48 participants (24 per group) is needed to detect a difference of 67 m on the 6-minute walk test (6MWT) between the two groups at a two-sided 0.025 significance level with 80% power, assuming that the standard deviation (SD) of the response variable is 150 m. The minimal clinically important difference (MCID) for the 6MWT is estimated to be 54-80 m using both distributional and discriminative methods. A sample size of 98 participants (49 per group) is needed to detect a difference of 5 points in the physical component score (PCS) between the 2 groups on the Short From-36 questionnaire (SF-36) with a two-sided 5% significance level and 80% power, assuming the SD of the response variable is 10 points.

The MCID in quality of life as measured by the PCS of the SF-36 is a change score of 5. We will use the larger sample of the two as the study size to ensure we have adequate power for both hypothesis tests. We will use an 'intention to treat' strategy in our primary analysis, however we adjusted our sample size upward to account for a planned 10% loss to follow-up to allow a secondary analysis using only participants with complete outcome data. We therefore plan to randomize 108 participants in the trial.

Procedures

In brief, the Principal Investigator (PI) and or study staff will be provided with the contact information and or medical record numbers of interested participants from referring clinicians or health care professionals at the University of Ottawa Heart Institute, The Ottawa Hospital, along with family health teams, general practices and cardiology clinics in the Champlain region of Ontario. Each participant will be asked to complete the following: 1) screening, 2) baseline, and 3) 12 week exercise training program with pre and post measures. The anticipated duration of participation will be 16 weeks. It is possible that the duration of participation may be greater or less than 16 weeks based on participants' schedule availability. Each component of the study is described in more detail below.

Recruitment and Screening

We will contact clinicians or health care professionals from the University of Ottawa Heart Institute, The Ottawa Hospital, along with family health teams, general practices and cardiology clinics in the Champlain region of Ontario to inform them of this study. These clinicians or health care professionals will identify and refer potentially eligible patients with persistent or permanent atrial fibrillation interested in participating in an exercise training study to Dr. Jennifer Reed and or a Research Coordinator (RC). Specifically, prospective research participants will be identified from clinicians or health care professionals at the University of Ottawa Heart Institute, The Ottawa Hospital, as well as partnering family health teams, general practices or cardiology clinics in the Champlain region of Ontario. If a patient expresses interest in participating in research to a heath care professional within their circle of care, this will be acknowledged as permission to contact the prospective participant, in order to provide them with study information and determine interest and eligibility. The PI and or RC may also screen for eligible patients using vOACIS, OIBEE or other relevant UOHI databases. The PI and or RC may also screen patients coming to Heart Institute clinics for eligibility. The PI and or RC will only contact patients who have given permission to be contacted by research to provide them with information about the study and determine if they are interested in participating. Interested individuals will be contacted by the PI and or RC who will prescreen patients for eligibility and arrange a visit to confirm eligibility and obtain written, informed consent. Other methods of advertising that may be used include newsletters directed at hospital staff and advertisements run in local newspapers and on local radio stations. Approved posters and or flyers may also be distributed to pharmacies, seniors' centers, community centers, recreation centers, cultural centers, churches, malls and libraries within the Champlain region. Secondary school students may volunteer to help with flyering in their community as part of the recruitment process. Additionally, the PI may have the opportunity to speak about her research surrounding exercise training in patients with atrial fibrillation on local TV and or radio stations. In this case, she may mention that recruitment is taking place for this study (OPPORTUNITY) and provide the email and or phone number of the RC. Furthermore, advertisements will be placed on the University of Ottawa Heart Institute's public website, social media and the University of Ottawa Heart Institute's Patient Alumni website and distribution list.

During the screening visit, the study staff will obtain participants' medical history, medication regime, age, sex, resting blood pressure and resting heart rate. Participants will also complete a cardiopulmonary exercise test on an electronically braked cycle ergometer (COSMED, USA) at the University of Ottawa Heart Institute Cardiac Diagnostic Centre or in the Track area of the Division of Prevention and Rehabilitation using a Carefusion Oxycon Mobile portable metabolic measurement system (p/n J143250). They will also undergo a medical assessment with a UOHI physician. Gender-related data will be collected retrospectively during one of the 1- to 10-year follow-up visits. Participants who have completed or have declined to participate in the 1- to 10-year follow-up visits will be contacted by phone and asked whether they would be willing to answer a set of questions related to their gender identity, relations and roles. Participants will be given a paper copy (in person during their follow-up visit or paper copy will be sent with a pre-paid envelope to their home address).

The study staff will record the participants' OHIP number to link their study information with the provincial health care database named Institute for Clinical Evaluative Sciences (IC/ES), to allow the study staff to track the use of health care services. Staff will use the IC/ES database to obtain data about emergency department visits, hospitalizations, day procedures and general/specialist visits. All linked data will be anonymized before being provided to us by IC/ES and all data will be analyzed and reported as group data, with no identifying information for any individual person reported or published. The OHIP number will be collected from the participants' medical chart using the University of Ottawa Heart Institute medical system. This information will be recorded in a password protected master list, located in the UOHI servers.

Baseline

During the baseline phase, participants will perform a 6-minute walk test. Study staff will extract demographic and clinical information from the patient chart regarding medications, cardiovascular health, co-morbidities (e.g. diabetes, arthritis, peripheral vascular disease, cerebrovascular disease, chronic obstructive pulmonary disease), and exercise training status. It will not be possible to extract this information for patients that do not come from UOHI or TOH, so they will be asked to provide these details or the referring clinician/clinic may provide this information providing the participant has given their consent to do so. Height, body weight, waist circumference, fat mass, heart rate and blood pressure will be measured using standardized procedures and information regarding participant demographics and menopausal status (of female participants only) will be collected. Participants will perform standard load tests to assess lower body muscular fitness. They will be fitted with a Holter monitor and given a wear-time journal for completion over a 24-hour period. Participants will complete questionnaires assessing quality of life, symptom burden, activity status, depression and anxiety, sleep apnea, and insomnia. Participants will then be provided with an Actigraph motion sensor, to be worn for 7 days. They will complete a FITT log over this period of time to assist with the verification of the data collected while the monitor was worn. They will also complete a 7-day symptom diary to record any symptoms related to atrial fibrillation and a 7 day sleep diary to record sleep patterns. All participants will be asked if they are willing to complete an additional cardiopulmonary exercise test at baseline on a cycle ergometer using a Carefusion Oxycon Mobile portable metabolic measurement system. Only the subset of participants who are willing to do this measure will complete it.

Intervention

Following the baseline phase, participants will be randomized in a 1:1 ratio to: 1) standard care + moderate-intensity continuous exercise training (to be completed in the University of Ottawa Heart Institute cardiovascular rehabilitation setting), or 2) standard care + high-intensity interval training using a blocked, stratified, random sequence that is computer-generated. Treatment assignments will be generated and placed in sealed, numbered envelopes to ensure concealment until baseline data are collected.

Participants will complete supervised exercise sessions that will take place in the Prevention and Rehabilitation Centre at University of Ottawa Heart Institute or in the *Goodlife* exercise facilities at The Ottawa Hospital – Civic Campus. Moderate-intensity continuous exercise training will follow cardiovascular rehabilitations guidelines: (1) warm-up for 5-10 minutes consisting of cardiovascular and or muscular endurance type activities at 20-35% of heart rate reserve for, rating of perceived exertion (RPE) of 6 to 11); (2) conditioning for 20-40 minutes at 40-85% heart rate reserve (or, RPE of 12 to 16); and, (3) cool down for 5-10 minutes at <60% heart rate reserve (or, RPE <12). Participants will attend on-site moderate-intensity continuous exercise training two times weekly for 12 weeks; they **will not attend educational workshops**. High-intensity interval training will include: (1) warm-up for 2 minutes at 50% of peak power output; (2) 2 x 8-minute interval training blocks (total: 16 minutes) of 30-seconds at 80-150% of peak power output interspersed with 30-seconds active recovery, four minutes of passive recovery will be permitted between the blocks; and (3) 1 minute cool down at 25% of peak power output after the last 30-second exercise bout. Participants will attend on-site high-intensity interval training two times weekly for 12 weeks. Blood pressure, heart rate and ratings of perceived exertion will be measured during the exercise sessions. Participants in the moderate-intensity continuous arm may undergo telemetry as is standard of care for cardiovascular rehabilitation. Pending the findings of telemetry, they may be asked to attend an additional medical appointment with a UOHI physician. Both the moderate-intensity continuous exercise and high intensity interval training arms will be encouraged to partake in moderate intensity aerobic exercise (RPE: 12-15) three to five additional times per week (aside from their two exercise sessions done at UOHI) for 30-60 minutes per session to accumulate approximately 200-400 total minutes of moderate intensity aerobic exercise per week. They will also be encouraged to do 2-3 strength training sessions throughout the week.

Follow Up Week 12

During or following week 12 of the intervention (post), body weight, waist circumference, fat mass, heart rate and blood pressure will be measured using standardized procedures and information regarding participant demographics and menopausal status (of female participants only) will be collected. Participants perform standard load tests to assess lower body muscular fitness. They will be fitted with a Holter monitor and given a wear-time journal for completion over a 24-hour period. Participants will complete questionnaires assessing quality of life (Short Form 36; University of Toronto Atrial Fibrillation Severity Scale), symptom burden (Canadian Cardiovascular Society Severity of Atrial Fibrillation Scale),functional capacity (Duke Activity Status Index), anxiety and depression (Hospital Anxiety and Depression Scale, insomnia (Insomnia Severity Index) and sleep apnea (STOP-BANG Sleep Apnea Questionnaire). Participants will also complete a 6-minute walk test.

Participants will again be provided with an Actigraph motion sensor, to be worn for 7 days, and complete a daily FITT log over the 7-day period. They will also complete the 7-day symptom diary to

record atrial fibrillation symptoms and a 7 day sleep diary to record sleep patterns at the conclusion of the study.

All participants will be asked if they are willing to complete an additional cardiopulmonary exercise test at follow up on a cycle ergometer using a Carefusion Oxycon Mobile portable metabolic measurement system. Only the subset of participants who are willing to do this measure will complete it.

Boxes marked with an ‘x’ show which procedures will occur at each study visit or during each study week.

			**Exercise Training**												**Follow Up**
**Visit**	**Screening**	**Baseline**	**1**	**2**	**3**	**4**	**5**	**6**	**7**	**8**	**9**	**10**	**11**	**12**	**Wk 26; Year 1 to year 10**
Length of time needed	60 mins	3-3.5 hrs	1-2 hrs	1-2 hrs	1-2 hrs	1-2 hrs	1-2 hrs	1-2 hrs	1-2 hrs	1-2 hrs	1-2 hrs	1-2 hrs	1-2 hrs	3-3.5 hrs	35 mins
Informed consent	x														
Medical history	x														
Age	x														
Sex (male/female)	x														
Height		x													
Weight		x												x	
Waist circumference		x												x	x
Fat mass		x												x	x
Resting blood pressure and heart rate	x	x	x	x	x	x	x	x	x	x	x	x	X	x	x
Exercise blood pressure and heart rate			x	x	x	x	x	x	x	x	x	x	x	x	x
Cardiopulmonary exercise test	x	x (if willing)												x (if willing)	
24- hour Holter monitor		x												x	
Muscular fitness (standard load tests)		x												x	
Physical activity levels (motion sensor)		x												x	x
7-day FITT log		x												x	x
7-day symptom diary		x												x	x
7-day sleep diary		x												x	x
6-minute walk test		x												x	x
Questionnaires		x												x	x
Exercise training sessions done onsite at UOHI			x	x	x	x	x	x	x	x	x	x	x	x	

Follow Up (Week 26; Year 1 to year 10)
Approximately 26 weeks and from year 1 to year 10 after completing the intervention, participants will be asked to return for a follow up visit. Body weight, waist circumference, fat mass, heart rate and blood pressure will be measured using standardized procedures and information regarding participant demographics and menopausal status (of female participants only) will be collected. Participants will complete questionnaires assessing quality of life (Short Form 36; University of Toronto Atrial Fibrillation Severity Scale), symptom burden (Canadian Cardiovascular Society Severity of Atrial Fibrillation Scale), functional capacity (Duke Activity Status Index), anxiety and depression (Hospital Anxiety and Depression Scale, insomnia (Insomnia Severity Index) and sleep apnea (STOP-BANG Sleep Apnea Questionnaire). Participants will also complete a 6-minute walk test.

Participants will be provided with an Actigraph motion sensor, to be worn for 7 days, and complete a daily FITT log over the 7-day period. They will also complete the 7-day symptom diary to record atrial fibrillation symptoms and a 7-day sleep diary to record sleep patterns. Depending on when participants are enrolled in the study, they may or may not be within the time frame to complete all of these visits.

Primary Outcome & Primary Measures (endpoints):
There will be two primary outcomes: 1) changes in quality of life measured by the Short Form 36 Health Survey Questionnaire (SF-36); and, 2) changes in exercise capacity measured by the 6 minute walk test (6MWT) over the 12 week intervention period.

Quality of life: will be measured using the widely used and thoroughly validated SF-36 questionnaire. It yields an 8-scale profile of functional health and well-being scores as well as psychometrically-based physical and mental health summary measures.

Exercise capacity: will be measured using the 6MWT. Patients will be instructed to walk as far as possible for 6 minutes. The 6MWT is highly reproducible, and 6MWT distance performance is highly correlated with objective measures including VO2peak and survival.

Secondary Measures
Secondary measures will include: 1) exercise adherence measured by accelerometer (and verified using a 7-day FIIT log; 2) activity status measured by the Duke Activity Status Index (DASI); 3) atrial fibrillation specific quality of life measured by the University of Toronto Atrial Fibrillation Severity Scale (AFSS); 4) symptom burden measured by the Canadian Cardiovascular Society Severity of Atrial Fibrillation (CCS-SAF) scale; 5) self-reported symptom frequency and severity measured by the 7-day symptom diary; 6) anxiety and depressive symptoms measured by the Hospital Anxiety and Depression Scale (HADS); 7) insomnia severity measured by the Insomnia Severity Index; 8) sleep apnea risk measured by the STOP-BANG Sleep Apnea Questionnaire; and 9) self reported sleep patterns measured by the 7-day sleep diary.

1. Exercise adherence: will be measured directly and by self-report. The Actigraph GT3X accelerometer will be used for direct measurement and worn over the right hip for a 7-day recording period. The activity monitor provides physical activity measurements including activity counts, intensity levels and energy expenditure. A FITT (frequency, intensity, time, type) log will be used to gather weekly self-reported data of exercise training over the course of the 12 weeks of study

participation. Minutes of moderate and vigorous intensity exercise training per week will be summed and used to assess moderate-intensity continuous exercise training and high-intensity interval training adherence.

2. Activity status: will be measured using the Duke Activity Status Index (DASI), a self-administered questionnaire comprising 12 questions that determines a persons' functional capacity. Higher scores represent greater functional capacity. The DASI is a validated measure that is highly correlated with objective measures (e.g. VO2peak).

3. Atrial fibrillation Specific Quality of life: will be measured using the University of Toronto Atrial Fibrillation Severity Scale (AFSS). The AFSS is a disease-specific quality of life measure used to capture subjective and objective ratings of disease burden in patients with atrial fibrillation. It consists of 19 items and assesses atrial fibrillation burden (4 items), health care utilization (4 items) and symptom severity (7 items). The AFSS has been tested according to standardized psychometric parameters of content validity, reliability and variability.

4. Symptom burden: will be measured using the Canadian Cardiovascular Society Severity of Atrial Fibrillation Scale (CCS-SAF). The CCS-SAF is a simple, concise, symptom-based severity scale to assess patient status. The CCS-SAF score is determined using 3 steps: 1) documentation of possible atrial fibrillation-related symptoms (palpitations, dyspnea, dizziness/syncope, chest pain, weakness/fatigue); 2) determination of symptom-rhythm correlation; and 3) assessment of the effect of these symptoms on patient daily function and quality of life. CCS-SAF scores range from 0 to 4, with highest values denoting severe impact of symptoms on quality of life and activities of daily living. The CCS-SAF has been validated in patients with atrial fibrillation.

5. Self-reported symptom frequency and severity: will be measured using the 7-day symptom diary. Symptoms attributed to atrial fibrillation are recorded on this diary as they occur. Date and time are noted along with the severity of each symptom; multiple pages will be available to accommodate patients with frequent symptoms. These symptoms will be recorded at baseline, and again at the end of the study.

6. Anxiety and depressive symptoms: will be measured using the Hospital Anxiety and Depression Scale (HADS). The HADS is a validated questionnaire, widely used in clinical settings and found to be a reliable instrument for detecting states of depression and anxiety, which is a common issue among patients with atrial fibrillation. It consists of two 7-point subscales of anxiety and depression.

7. Insomnia severity: will be measured using the Insomnia Severity Index. It is comprised of 7 questions used to assess the nature, severity and impact of insomnia. It has been demonstrated to be a reliable measurement tool and content validity was formally demonstrated via principal component analysis.

8. Sleep apnea risk: will be measured by the STOP-BANG Sleep Apnea Questionnaire. This is a 7-item validated questionnaire used to evaluate the risk of obstructive sleep apnea in patients. Sleep apnea is a common issue among patients with atrial fibrillation. Patients deemed at high risk for sleep apnea may be referred for a sleep assessment by Dr. Andrew Pipe or Dr. Heather Tulloch.

9. Self reported sleep patterns: will be measured using a 7-day sleep diary. This will be used to capture any sleep problems and measure any progress in improving sleep. Details regarding sleep timing, quality, naps, alcohol consumption and sleep medication will be collected over a 7-day period.

10. Heart rate control: measured using 24-hour Holter ECG recordings and ECG recordings at each CPET.

11. Muscular fitness**:** measured using standard load tests. This test involves the completion of repetitions of leg press exercises, at a defined resistance of 40 kg at a cadence of 22 repetitions per minute (set by a metronome). The maximum number of repetitions done before falling behind the required cadence will be recorded.

12. Exercise capacity: measured using a gold standard symptom-limited, cardiopulmonary exercise test (CPET) on an electronically braked cycle ergometer using a ramp protocol. Gas exchange will be monitored continuously; the highest rate of oxygen uptake achieved (i.e. peak VO_2 in mL/kg/min) during the last minute of the CPET will represent exercise capacity. All participants will be asked if they are willing to complete this measure at baseline and follow up and only those who are willing will complete it.

Additional Measures
Cardio-respiratory fitness (measured at baseline only): will be evaluated during a symptom-limited, cardio-pulmonary exercise test on an electronically braked cycle ergometer using a ramp protocol. Peak power output will be evaluated during this exercise test and used for moderate-intensity continuous exercise training and high-intensity interval training exercise prescription. Whole-body VO_2 will be measured using expired gas samples.

Body weight: will be measured using a digital scale to the nearest 0.1 kilogram during the baseline, intervention and follow up phases.

Waist circumference: will be measured using a tape measure during the baseline, intervention and follow up phases.

Fat mass: will be measured using bioelectrical impedance analysis during the baseline, intervention and follow up phases. Participants will be asked to follow the pretesting guidelines for bioelectrical analysis testing:

- No eating or drinking within 4 hours of the test
- No moderate or vigorous exercise within 12 hours of the test
- Void urine completely within 30 minutes of the test
- Abstain from alcohol consumption within 48 hours of the test
- Do not ingest diuretics, including caffeine before the assessment unless they are prescribed by a physician
- If you are in a stage of your menstrual cycle during which you perceive you are retaining water, postpone testing (female participants only)

Resting and exercise blood pressure: will be measured using a sphygmomanometer during the baseline and intervention as well as during the exercise training sessions.

Resting and exercise heart rate: will be measured manually or using polar heart rate monitors during the baseline and intervention phases as well as during the exercise training sessions.

Rating of perceived exertion: will be measure during the exercise tests and training sessions.

Gender-related data: will be measured using a set of questions related to gender identity, relations and roles. Participants will be given a paper copy which can be completed in person during their visit or alternatively it will be sent to their home address using a pre-paid envelope.

Analyses of Results
Repeated measures mixed models analyses will be performed to examine changes in primary and secondary outcomes.

Risks, Benefits and Usual Standard of Care
The usual standard of care for patients with persistent or permanent atrial fibrillation includes the following steps: 1) to make sure patient is well rate controlled, 2) to make sure patient is anti-coagulated if appropriate, and 3) if well rate controlled and anti-coagulated, then patient is discharged to family doctor. If patients choose to take part in this study, each participant will be asked to also complete the following: 1) screening, 2) baseline, and 3) 12-week exercise training program with repeated measures. These aspects of the study have been described in detail in the study protocol. No changes will occur to usual standard of care.

Risks of Participant
Risks for usual standard of care:
Standard of care which consists of rate or rhythm control as well as anti-coagulation control significantly reduces the risk of stroke and decline in ventricular performance in patients with uncontrolled persistent or permanent atrial fibrillation. No changes will occur to usual standard of care.

Risks for research participants:
Participation in this study requires that participants perform exercise tests and training sessions. The risk of cardiovascular events during exercise testing varies directly with the incidence of cardiovascular disease. The risk of exercise testing is low, with approximately 6 cardiac events per 10,000 tests (0.06%). This is comparable to the usual care that patients in cardiovascular rehabilitation programs would receive. Recent studies have reported a rate of 1 cardiac arrest per 116,906 patient-hours, 1 myocardial infarction per 219,970 patient-hours, 1 fatality per 752,365 patient-hours, and 1 major complication per 81,670 patient-hours in a cardiovascular rehabilitation setting. It should be noted that these rates are low and likely due to the fact that patients in these studies were screened and exercised in a medically supervised setting equipped to handle emergencies. This is the case of the University of Ottawa Heart Institute, Division of Prevention and Rehabilitation.

The commonly reported side effects that may result from exercise testing and training sessions may include palpitation, chest pain, shortness of breath, headache, nausea and/or fatigue.

Participants may find the questionnaires regarding quality of life (Short Form 36, University of Toronto Atrial Fibrillation Severity Scale), symptom burden (Canadian Cardiovascular Society Severity of Atrial Fibrillation Scale), functional capacity (Duke Activity Status Index), anxiety and depression (Hospital Anxiety and Depression Scale, insomnia (Insomnia Severity Index) and sleep apnea (STOP-BANG Sleep Apnea Questionnaire) distressing.

Monitoring and Reporting of Adverse Events/Serious Adverse Events
The following definitions have been approved as part of Good Clinical Practice in Research:

Unanticipated Problem
An unanticipated problem is defined as any incident, experience, or outcome that meets all of the following criteria:

Unexpected (in terms of nature, severity, or frequency) given (a) the research procedures that are described in the protocol-related documents, such as the REB-approved research protocol and informed consent document, or the Investigator Brochure (IB) or product monograph (PM); and (b) the characteristics of the research participant population being studied; and

Related or possibly related to participation in the research (possibly related means there is a reasonable possibility that the incident, experience, or outcome may have been caused by the [investigational product(s)] or procedures involved in the research); and

Suggests that the research places research participants or others at a greater risk of harm (including physical, psychological, economic, or social harm) than was previously known or recognized.

Adverse event
An adverse event is defined as any untoward medical occurrence in a research participant administered an investigational product and which does not necessarily have a causal relationship with this product. An AE can therefore be any unfavorable and unintended sign (including an abnormal laboratory finding, for example), symptom, or disease temporally associated with the use of an investigational product, whether or not related to the investigational product.

Serious Adverse Event
To ensure no confusion or misunderstanding of the difference between the terms "serious" and "severe," which are not synonymous, the following note of clarification is provided:

The term "severe" is often used to describe the intensity (severity) of a specific event (as in mild, moderate, or severe myocardial infarction); the event itself, however, may be of relatively minor medical significance (such as severe headache).

This is not the same as "serious," which is based on patient/event outcome or action criteria usually associated with events that pose a threat to a patient's life or functioning.

Seriousness (not severity) serves as a guide for defining regulatory reporting obligations.

After reviewing the various regulatory and other definitions in use or under discussion elsewhere, the following definition is believed to encompass the spirit and meaning of them all:

A serious adverse event (experience) or reaction is any untoward medical occurrence that at any dose:

- results in death;
- is life-threatening;
- requires inpatient hospitalization or prolongation of existing hospitalization;
- results in persistent or significant disability/incapacity; or
- is a congenital anomaly/birth defect.

The term "life-threatening" in the definition of "serious" refers to an event in which the patient was at risk of death at the time of the event; it does not refer to an event which hypothetically might have caused death if it were more severe.

Medical and scientific judgment should be exercised in deciding whether expedited reporting is appropriate in other situations, such as important medical events that may not be immediately life-threatening or result in death or hospitalization but may jeopardize the patient or may require intervention to prevent one of the other outcomes listed in the definition above. These should also usually be considered serious.

Examples of such events are intensive treatment in an emergency room or at home for allergic bronchospasm; blood dyscrasias or convulsions that do not result in hospitalization; or development of drug dependency or drug abuse.

All unanticipated problems/adverse events/serious adverse events will be recorded on the case report form (CRF) and in the source documents at the site of testing. A log for all unanticipated problems/adverse events/serious adverse events occurring in the study will be kept. The details of any unanticipated problems/adverse events/serious adverse events at each study visit, using the protocol-defined terminology, will be recorded.

The PI and or RC will educate the participants about unanticipated problems/adverse events/serious adverse events as well as the importance of reporting them to the study coordinator at study visits.

Anyone within the research team who becomes aware of unanticipated problems/adverse events/serious adverse events will report this to thePI. The PI will assess the unanticipated problems/adverse events/serious adverse event and provide the research participant with appropriate medical care as applicable. The PI will report to the REB within 7 days only the local adverse events/serious adverse events that are deemed to be unanticipated problems.

The monitoring of these unanticipated problems/adverse events/serious adverse will be made in accordance with the Ottawa Health Sciences Network Research Ethics Board’s SOP 404.001 Ongoing REB Review Activities including the SOP 404 Adverse Event Reporting Requirements addendum.

Risks of Insurability

All study staff members will take all reasonable steps to ensure participant research information is kept confidential. Should someone not involved in the research find out about the identity(ies) of individual participants, there is a possibility that this could affect one's insurability under certain policies of insurance, depending on the exclusions in such policies.

Benefits of Participant Participation

Participants may not receive any direct benefit from their participation in this study. Upon completion of the study, participants will receive a summary of their fitness measurements.

Withdrawal Criteria

Participants have the right to withdraw from the study at any point in time. Staff conducting the study has the right to withdraw participants from this study for any of the following reasons:

- The investigator feels it is in the participant's best interest.
- Participant needs medication/treatment that would interfere with the study.
- Participant does not follow the study staff's instructions.

Confidentiality/Privacy

All Personal Health Information (PHI) and Personal Identifying Information (PII) will be kept confidential, unless release is required by law. Release of PHI/PII information will only be allowed if it is legally required by law. For audit purposes only, representatives of the Ottawa Health Sciences Network Research Ethics Board (OHSN-REB) and the University of Ottawa Heart Institute may review participants' records under the supervision of Dr. Jennifer Reed's staff.

Results of research studies should be shared, to ensure participants are always provided with the best possible care. Therefore, results from this study may be presented at scientific conferences and/or published in journals but participants' will not be identifiable in any publications or presentations.

All participants will be assigned a study ID number once informed consent in obtained. The study ID will serve as the only identifier used on all study related documents. Informed consent will occur at the time of recruitment once verification of eligibility has been completed. The master list will be maintained by study staff and stored in a separate file from the coded study dataset on a password protected computer. The master list will be in a password protected file stored separately from all other study files; the master file will be password protected and only individuals directly involved with the study (e.g., PI or designate) will have access to this file. The participant identifiers to be stored in the master list include full name and contact information (e.g., phone number, email, address). The University of Ottawa Heart Institute and the Health Sciences Network Research Ethics Board (OHSN-REB) will have access to the records for audit purposes.

A separate database will be kept that contains study ID numbers along with personal identifiers. The database containing descriptive information (e.g., height, weight, etc.) will only have study ID numbers as identifiers. Case Report forms will only have study ID numbers used as identifiers, all other personal information (e.g., name) will not be collected on these forms. Participants will be reminded

not to write their name on any forms. Please refer to the Case Report Form (CRF) for personal health information to be collected.

No identifying information will leave the University of Ottawa Heart Institute. All information that leaves the University of Ottawa Heart Institute will be coded with an independent study number that will be used throughout the study on all of the study records. The Master List which links participants' name and the independent study number will only be accessible by Dr. Jennifer Reed and/or her staff. The link and study files will be stored separately and securely.

Record Keeping/Document Management

The research staff will collect the case report forms and questionnaires. Both paper and electronic records will be provided to Dr. Reed and her staff. All paper records will be stored in a locked filing cabinet and office. All electronic records, including the Master List, will be stored on a secure internal hospital server and password protected, only accessible by Dr. Reed and/or her staff. No identifiable information will be stored on any mobile devices (laptops, USB keys, CDs, DVDs, etc.). Research files will be kept for a period of 10 years after the study has been completed, as required by law. At the end of the retention period, all paper records will be disposed of in confidential waste for shredding and all electronic records will be securely deleted.

Data Handling

All data will only be accessible by Dr. Jennifer Reed and/or co-investigators or collaborators or staff.
