# Supplementary material for: Effect of High-Intensity Interval Training in Patients With Atrial Fibrillation: A Randomized Clinical Trial
Source: JAMA Netw Open. 2022 Oct 31;5(10):e2239380. doi: 10.1001/jamanetworkopen.2022.39380 (PMC9623436; doi:10.1001/jamanetworkopen.2022.39380)
Supplement: Supplement 2. — Data Sharing Statement [file jamanetwopen-e2239380-s002.pdf]

Data Sharing Statement

Reed. Effect of High-Intensity Interval Training in Patients With Atrial Fibrillation. *JAMA Netw Open*. Published October 31, 2022. doi:10.1001/jamanetworkopen.2022.39380

Data
Data available: No

Additional Information
Explanation for why data not available: A data sharing agreement can be requested to our legal team.
